# Enhancing lexical tone learning for second language speakers: effects of acoustic properties in Mandarin tone perception

**DOI:** 10.3389/fpsyg.2024.1403816

**Published:** 2024-08-21

**Authors:** Meng Cao, Philip I. Pavlik, Gavin M. Bidelman

**Affiliations:** ^1^Optimal Learning Lab, Department of Psychology, Institute for Intelligent Systems, University of Memphis, Memphis, TN, United States; ^2^Department of Speech, Language, and Hearing Sciences, Indiana University, Bloomington, IN, United States; ^3^Program in Neuroscience, Indiana University, Bloomington, IN, United States; ^4^Cognitive Science Program, Indiana University, Bloomington, IN, United States

**Keywords:** lexical tones, Mandarin tones, second language acquisition, tone exaggeration, acoustic properties, fundamental frequency, duration

## Abstract

Understanding the challenges faced by second language (L2) learners in lexical tone perception is crucial for effective language acquisition. This study investigates the impact of exaggerated acoustic properties on facilitating Mandarin tone learning for English speakers. Using synthesized tone stimuli, we systematically manipulated pitch contours through three key modifications: expanding the fundamental frequency (F0), increasing F0 (female voice), and extending the overall duration. Our objectives were to assess the influence of F0 expansion, higher F0, longer duration, and varied syllables on Mandarin tone learning and generalization. Participants engaged in a non-adaptive trial-by-trial tone identification task. Mixed-effects logistic regression modeling was used to analyze accuracy across learning phases, acoustic factors, and tones. Findings reveal improvements in accuracy from training to testing and generalization phases, indicating the effectiveness of perceptual training to tone perception for adult English speakers. Tone 1 emerged as the easiest to perceive, while Tone 3 posed the most challenge, consistent with established hierarchies of tonal acquisition difficulty. Analysis of acoustic factors highlighted tone-specific effects. Expanded F0 was beneficial for the identification of Tone 2 and Tone 3 but posed challenges for Tone 1 and Tone 4. Additionally, longer durations also exhibited varied effects across tones, aiding in the identification of Tone 3 and Tone 4 but hindering Tone 1 identification. The higher F0 was advantageous for Tone 2 but disadvantageous for Tone 3. Furthermore, the syllable *ma* facilitated the identification of Tone 1 and Tone 2 but not for Tone 3 and Tone 4. These findings enhance our understanding of the role of acoustic properties in L2 tone perception and have implications for the design of effective training programs for second language acquisition.

## Introduction

Lexical tone perception is essential when learning tonal languages such as Mandarin Chinese. However, acquiring lexical tones has been identified as a challenging task for adult second language (L2) learners, particularly for those from non-tonal native language backgrounds (e.g., L1 English speakers; Kiriloff, 1969; Bluhme and Burr, 1972; Shen, 1989; Francis et al., 2008; Shen and Froud, 2016, 2019; Wang et al., 1999). Understanding the factors that contribute to these challenges is essential for developing effective training methodologies to support L2 learners in acquiring lexical tones.

Research indicates that the perception and discrimination of tones are heavily influenced by a listener’s language experience and their degree of familiarity with lexical tones (Xu et al., 2006; Francis et al., 2008; Chandrasekaran et al., 2014; Bidelman and Lee, 2015). Indeed, non-tonal language speakers are less sensitive to tonal variations, whereas native speakers of tonal languages exhibit greater attention to tonal features (Hallé et al., 2004; Chandrasekaran et al., 2009). The slow learning of tonal features, potentially linked to reduced sensitivity to tones, has prompted researchers to explore practical solutions for learners. In language acquisition, one approach to improve perceptual contrasts and assist learners in recognizing essential acoustic cues is the utilization of exaggerated stimuli (Merzenich et al., 1996; Tallal et al., 1996; McCandliss et al., 2002; Shih et al., 2010). For instance, a study on Japanese speakers learning to differentiate [*r*] and [*l*] contrasts demonstrated the effectiveness of using exaggerated stimuli in adaptive training (McCandliss et al., 2002).

Although the use of exaggerated stimuli has shown promise in language learning, its application in the context of lexical tone acquisition remains largely unexplored. Specifically, the role of acoustic properties in the challenges of tone perception and the potential of exaggerated tone stimuli to simplify this process for L2 learners are not well understood. Therefore, the present study investigates the effects of acoustic properties of lexical tones on tone perception for adult English speakers with Mandarin Chinese as the target language. By deliberately exaggerating specific acoustic properties of Mandarin tones, we aimed to identify which acoustic modification makes tone perception easier for L2 learners. The findings of this study could enable instructors to adjust the difficulty level of tone stimuli by manipulating their acoustic properties, thereby facilitating a progressive adjustment of difficulty. Furthermore, these findings could contribute to the development of effective training regimens, thereby enhancing the acquisition of lexical tones for L2 learners.

### Acoustic properties of Mandarin lexical tones

Mandarin Chinese is a tone language that uses contrastive pitch patterns (lexical tones) to convey word meaning at the syllable level. To recognize a spoken Mandarin word, listeners need to perceive both the syllable (e.g., consonants, vowels) and tone components of words to jointly determine the semantic value (Liu et al., 2011; Wiener and Lee, 2020). For example, syllable /ma/ in Tone 1 means “mother”; /ma/ in Tone 2 means “hemp”; /ma/ in Tone 3 means “horse”; /ma/ in Tone 4 means “to scold” (Liu et al., 2011).

**Figure 1:**
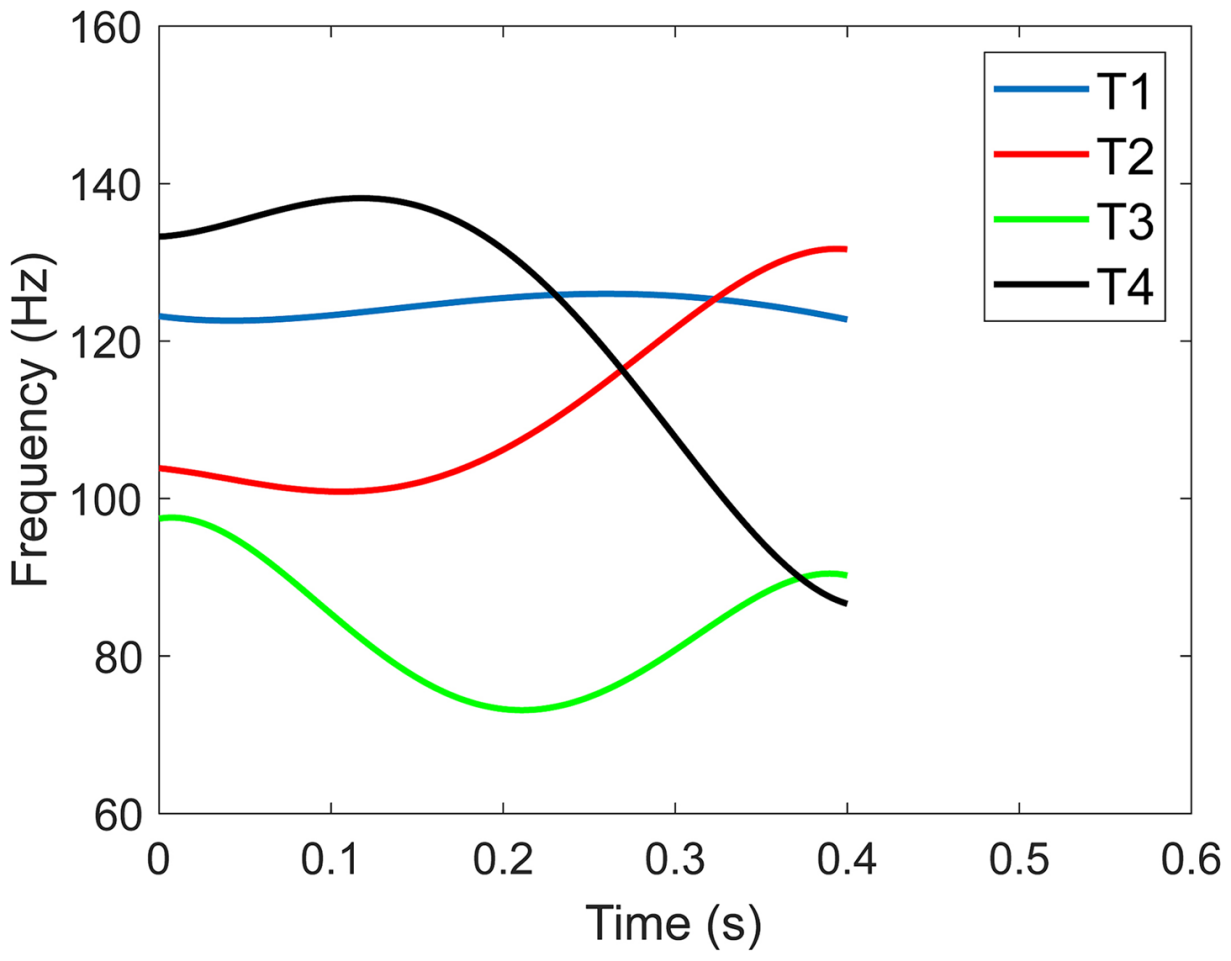
Synthesized F0 contours of the four Mandarin tones of male speaker at 400 ms.

The primary acoustic property that differentiates lexical tones is the fundamental frequency (F0) or pitch contour (Howie, 1976; Blicher et al., 1990; Whalen and Xu, 1992; Moore and Jongman, 1997; Xu, 1997; Peng, 2006; Liu et al., 2011; Wiener and Lee, 2020). F0 is the vibratory rate of the vocal folds that is perceived as the pitch of a speaker (Liu, 1924; Shih et al., 2010). Even though there are some variations in natural tone productions due to individual speaker differences (Lin, 1965; Xu, 1997; Chandrasekaran et al., 2010), the general pitch patterns of the four Mandarin tones are independent of the speaker (see Figure 1 which shows the synthesized F0 contours of the four tones produced by a male speaker with the duration of 400 ms): Tone 1 is a high-level tone that has a relatively flat F0 contour; Tone 2 is a rising tone that traverses from low to high F0; Tone 3 is a dipping tone whose F0 first falls then rises in a lower register; Tone 4 is a falling tone that starts with a high F0 value then falls rapidly.

According to the results of multidimensional scaling (MDS) analysis of tonal dissimilarity judgments, linguistic pitch patterns have three primary perceptual dimensions (height, direction, and contour) which serve as cues to tonal identification (Gandour, 1983). Even though listeners from various language backgrounds (e.g., English and Mandarin) use the same number of dimensions, they exhibit differences in the relative significance they assign to specific dimensions (Chandrasekaran et al., 2007). For example, English listeners pay more attention to the F0 height (average F0), whereas native Mandarin speakers pay more attention to F0 direction/contour (i.e., level, rising, or falling) (Gandour, 1983; Gottfried and Suiter, 1997; Xu et al., 2006; Chandrasekaran et al., 2007, 2010; Francis et al., 2008). The perceptual saliency of different dimensions is also influenced by learners’ language experience. In non-tonal languages like English, F0 height plays a more prominent role in conveying speaker-specific information, such as indicating questions or emphasizing certain words, but it is not used for distinguishing lexical meaning. Whereas for Mandarin Chinese, distinct F0 contours/directions are crucial in determining lexical content regardless of the variations in F0 height across different speakers (e.g., female voice has a higher pitch than male voice; Gandour, 1983; Chandrasekaran et al., 2010). Therefore, for L2 learners, the crucial aspect of acquiring tones lies in redirecting their focus from the F0 height to the F0 direction/contours. It is essential for learners to develop the skill to distinguish different F0 contours that characterize each tone.

In addition to F0 contour, duration and amplitude are considered secondary acoustic properties of Mandarin tones (Whalen and Xu, 1992; Liu et al., 2011; Wiener and Lee, 2020). Even though there are differences among the four tones regarding amplitude (Tone 3 has the lowest amplitude and Tone 4 the highest; Lin, 1965) and duration (Tone 3 has the longest duration, followed by Tone 2, Tone 1, with Tone 4 having the shortest duration. Xu, 1997), they are not the defining feature that distinguishes tones (Howie, 1976). L2 listeners rely more on duration when making a distinction between Tone 2 and Tone 3 while native Mandarin listeners do not (Chang, 2011). However, the reliability of duration as a distinguishing feature is questionable, particularly when L2 learners are exposed to tone stimuli produced by various talkers in different speech contexts. In such cases, the duration of a specific tone can vary widely, making it an inconsistent and unreliable cue for distinguishing between tones. Similarly, amplitude can also vary significantly based on an individual speaker’s speaking style and the speaking context. This variability can make amplitude, like duration, an unreliable cue for identifying tones, especially for L2 learners. Instead, L2 learners might benefit by focusing more on recognizing the F0 contours of the tones, which provide a more consistent and reliable basis for tone perception and learning.

### Acoustic modifications

One way to attract L2 learners’ attention to F0 direction/contours is by utilizing exaggerated stimuli to make this feature more salient. An area of research that supports this idea is speech hyperarticulation. In daily conversation, speakers modify their speech style and use a more “exaggerated” approach to enhance intelligibility in various speech contexts (Summers et al., 1988; Tupper et al., 2021). This speech modification also happens in tonal languages. For example, research has shown that in infant-direct speech (IDS, a speech style when talking to an infant; Kuhl et al., 1997; Burnham et al., 2002), Mandarin or Cantonese tones are hyperarticulated with a higher F0, longer duration, and an expanded F0 range or tone space (defined by the area formed by F0 onset/offset plots of different tones) compared to adult-directed speech (ADS; a speech style when talking to an adult; Grieser and Kuhl, 1988; Liu et al., 2007; Rattanasone et al., 2013). The increased F0 was also found in Lombard speech (a speech style in noisy environments; Zhao and Jurafsky, 2009; Tang et al., 2017). In foreign language instruction settings or teaching settings (a speech style when speaking to/teaching a non-native speaker), Mandarin tones exhibited an overall expanded F0 range and longer duration compared to natural speech (Papouˇsek and Hwang, 1991; Zhao and Jurafsky, 2009; Tang et al., 2017; Han et al., 2019). Furthermore, Mandarin tones produced in clear speech, also known as “clarified speaking style” in which each word is carefully articulated to maximize intelligibility, have a longer duration and increased intensity compared to plain, conversational speaking style (Tupper et al., 2021).

An expanded F0 range and tone space, which enhances the contrast of Mandarin tone categories, can be considered a code-based modification. This type of phoneme-specific change is critical for distinguishing one word from another in speech and, therefore, can be used to aid speech intelligibility (Kuhl et al., 1997; Rattanasone et al., 2013; Wedel et al., 2018; Tupper et al., 2021). Conversely, increased F0, intensity, and overall duration can be considered signal-based modifications. These modifications enhance the entire speech signal and do not depend on language properties, serving to attract listeners’ attention or convey the positive affect of the speaker (Kuhl et al., 1997; Uther et al., 2007; Tupper et al., 2021). Even though tone hyperarticulation is observed in different speech contexts, little research (discussed below) has investigated the use of such modifications to tone language training and how these enhancements could influence tone acquisition in L2 speakers.

### Training paradigms

A substantial body of research has demonstrated that auditory training on lexical tones can significantly help L2 learners to overcome the difficulty of tone perception (Wang et al., 1999, 2003; Francis et al., 2008; Shih et al., 2010; Liu et al., 2011; Zhao and Kuhl, 2015; Reetzke et al., 2018). Within such training, the design of training materials and the sequence of their presentation are both vital components. For example, High Variability Phonetic Training (HVPT) emphasizes the diversity of training materials but does not specifically focus on the sequencing of these materials. HVPT has proven effective in enhancing lexical tone learning, particularly when the tone stimuli are highly variable, such as those produced by different speakers in a range of phonetic contexts (Wang et al., 1999, 2003; Perrachione et al., 2011; Sadakata and McQueen, 2014; Li et al., 2016; Zhang et al., 2018; Dong et al., 2019; Wiener and Lee, 2020). However, a limitation of HVPT is that its effectiveness varies based on the learner’s tone perceptual aptitude, with high variability training impeding tone learning in low-aptitude individuals despite benefiting those with high aptitude (Sadakata and McQueen, 2014).

In contrast, adaptive training can address individual variability by adjusting the difficulty of items based on learners’ performance and has proven its effectiveness in tone learning (Shih et al., 2010; Li et al., 2016). Like HVPT, adaptive training also exposes learners to tone stimuli with a wide range of acoustic features. However, previous adaptive training methods indirectly manipulated acoustic properties to adjust difficulty, and the precise influence of these properties on tone perception and learning remains unexplored. For example, in an adaptive training study by Shih et al. (2010), native speakers were asked to produce tones at 11 different speaker-listener distances. The study then adjusted the difficulty of the tone stimuli based on these distances. It was hypothesized that tones produced from a greater distance would be more exaggerated and clearer, and therefore easier to perceive, while those produced at a closer distance would be softer, reduced, and consequently more difficult to perceive. Even though this approach addresses the varying difficulty levels of tone stimuli, it does not assess controlled variation in stimulus properties, leaving gaps in our understanding of how modifying acoustic properties of the speech material impacts the learning process. Furthermore, a better understanding of these influences could help design more precise adaptive training algorithms by effectively manipulating acoustic properties to adjust item difficulty.

### The present study

Addressing the gap in research regarding the influence of acoustic properties on the difficulty of learning lexical tones, our study explores whether modifications to these properties can aid in easier tone perception and learning. Specifically, we implement three key modifications to tones during the perceptual learning process: expanding the F0 range, increasing the F0, and lengthening the overall duration.

In our training paradigm, we employed HVPT, a method recognized for its effectiveness in fostering robust phonetic categorization (Logan et al., 1991; Lively et al., 1993). To increase the variability of tone stimuli, we synthesized tones with varying levels of F0 expansion, different durations, speaker sexes (our 3 key factors), and syllables, thereby creating a diverse and highly variable set of stimuli varying over 4 factors. We used non-adaptive trial-by-trial training (necessary to measure difficulty without a confound of adaptive selection) with corrective feedback after each response. Each trial consisted of auditory tone identification where learners labeled the tone from among four options. This method was used because laboratory training paradigms ubiquitously utilize trial-by-trial feedback to teach L2 speech categories (Lively et al., 1993; Bradlow et al., 1999; Tricomi et al., 2006; Zhang et al., 2009; Lim and Holt, 2011). The forced-choice procedure and immediate feedback direct participants’ attention to category-relevant acoustic cues so that they can form new phonetic categories (Jamieson and Morosan, 1986; Lively et al., 1993; Roelfsema et al., 2010; Reetzke et al., 2018). Initially, participants engaged in training trials focused on a specific level of a certain acoustic property (e.g., duration, F0 expansion, etc.). Their performance was then assessed using both the initial training stimuli and new stimuli, which incorporated a different level of the same acoustic property. Importantly, to aid in learning and evaluation, feedback was provided during all training and testing trials.

Additionally, research indicates that younger adults typically outperform older adults in lexical tone learning (Wang et al., 2017), and that English-speaking musicians demonstrate superior tone perception compared to non-musicians (Schön et al., 2004; Alexander et al., 2005; Marques et al., 2007; Wong et al., 2007; Chandrasekaran et al., 2009; Bidelman and Alain, 2015; Zhao and Kuhl, 2015). Therefore, we considered both the age and music training experience of L2 learners in our analyses.

We evaluated participants’ performance in tone identification across various factors: F0 expansion, F0 height, duration, and syllables. Our hypothesis was that modifications in F0 and duration would significantly influence tone learning. More specifically, vertically expanding the F0 contours (in frequency) which enhances the contrasts among Mandarin tone categories could help learners distinguish tones, thereby promoting tone perception and learning. Additionally, higher frequency (as in a female voice) and longer duration which enhance the entire speech signal, may also benefit Mandarin tone perception and learning. Furthermore, the inclusion of different syllables aimed to increase tone variability could also potentially have varying impacts on the acquisition of tone categories.

## Materials and methods

### Participants

Participants were recruited through the Amazon Mechanical Turk (MTurk) web service. To be eligible, participants needed to be over the age of 18, reside in Canada or the US, and have completed 100 MTurk tasks previously with 95% acceptance. These inclusion restrictions helped ensure our subject pool consisted of quality “workers.” We also required the participants to have little knowledge of Mandarin tones, and no (self-reported) hearing problems. The study was approved by the Institutional Review Board of the University of Memphis. Electronic consent was obtained from all participants. *N* = 325 MTurkers finished the experiment. 35 participants were excluded since their performance was below chance (< 25% correct).

A final sample of *N* = 290 participants was included in the analysis (131 female, 159 male). Age ranged across the lifespan: 10.2% were between 18 and 25 years old, 34.6% were 26–34, 45.4% were 35–54, and 7.8% were 55–64 years old. Only 2.0% were more than 65 years old. The survey question for education level showed that 12.7% had a high school diploma or GED, 42.0% had some college, 38.5% had a 4-year college degree or a bachelor’s degree, and 6.8% had a graduate degree. The survey question about music ability showed that 62.0% had no formal music training, 8.8% had ≤1 year of music training, 16.6% had 2–5 years of music training, and 12.7% had >5 years of music training.

### Stimuli

We adopted the Mandarin tone synthesis approach as described in Krishnan et al. (2010). We generated iterated rippled noise (IRN) stimuli in MATLAB, featuring dynamic F0 contours with pitches that vary over time (see *Supplementary material* for MATLAB code). The synthesis parameters for these F0 contours were derived from natural Mandarin speech, using 4th-order polynomial equations (Xu, 1997; Xu et al., 2006; Krishnan et al., 2010). Given that Tone 2 and Tone 3 are often confusable for both native and non-native listeners due to their similar pitch contours (Kiriloff, 1969; Chuang et al., 1972; Gottfried and Suiter, 1997; Reetzke et al., 2018), we made a specific modification to the equation for Tone 3. This adjustment aimed to lower the turning point of Tone 3, thereby enhancing its distinction from Tone 2. While there are various methods to differentiate between Tone 2 and Tone 3, such as altering the timing of the turning point (Shen and Lin, 1991), we did not further optimize this adjustment as such nuances were not the primary focus of our study.

#### F0 expansion

**Figure 2:**
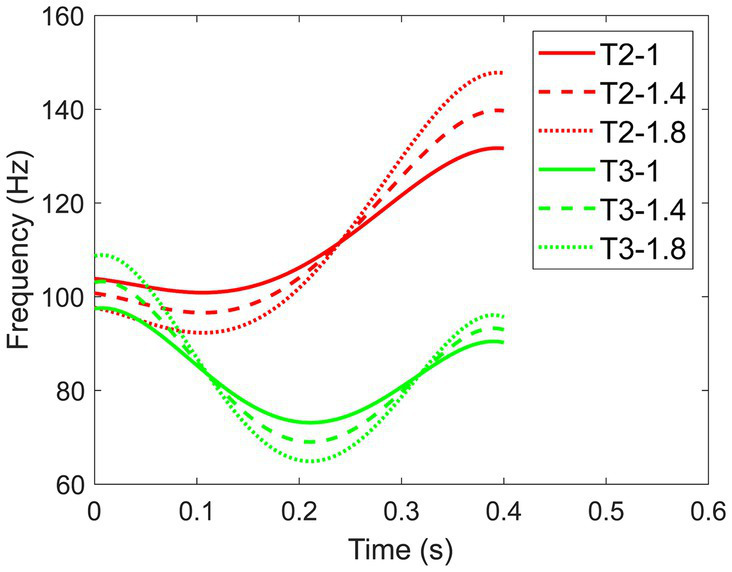
F0 contours of Tone 2 and Tone 3 at three F0 expansion levels (800 ms duration).

The expansion of the F0 range was achieved by stretching the F0 contour to make the four tones more contrastive in frequency. We employed Equation 1 to compute varying levels of expanded F0. Here, *t_n_* represents the adjusted frequency of the pitch of the *n^th^* tone, while *T_n_* represents the original frequency of the pitch. $\bar{T_{n}}$ denotes the mean frequency of the pitch, and *α* represents the expansion factor. *n* indicates which tone it is. The calculation of expanded F0 involved determining the difference between the original frequency of the pitch and the mean frequency. This difference was then scaled by the expansion factor and added back to the mean frequency. The multiplier (*α*) plays a crucial role in determining the extent of F0 expansion. When *α* = 1, *t_n_* was the regular F0 pattern. For *α* = 1.4, *t_n_* denoted a moderately expanded F0, resulting in the highest point of the pitch contour being 1.4 times higher and the lowest point 1.4 times lower. Similarly, for *α* = 1.8, *t_n_* embodied the widely expanded F0 (see Figure 2, the example F0 contours of Tone 2 and Tone 3).


(1) $$t_{n}=\left(T_{n}-\bar{T_{n}}\right)\times \alpha +\bar{T_{n}}\;\left(n=1,2,3,4\right)$$

#### Sex

The height difference of F0 was created by having tones produced by two different simulated sexes: male and female. While most previous research mainly used male voices, we used both male and female voices to increase the variety of stimuli and ecological validity. Additionally, females typically exhibit a higher frequency compared to the male voice, aligning with types of acoustic modifications observed in Infant-Directed Speech (e.g., Rattanasone et al., 2013) and Lombard speech (e.g., Tang et al., 2017). To achieve this higher frequency in our study, we applied a multiplier of 2 to the equations for male voices. This adjustment ensured that the fundamental frequency (F0) of the female voice (mean frequency is 220 Hz) was twice as high as that of the male voice (mean frequency is 110 Hz).

#### Duration

**Figure 3:**
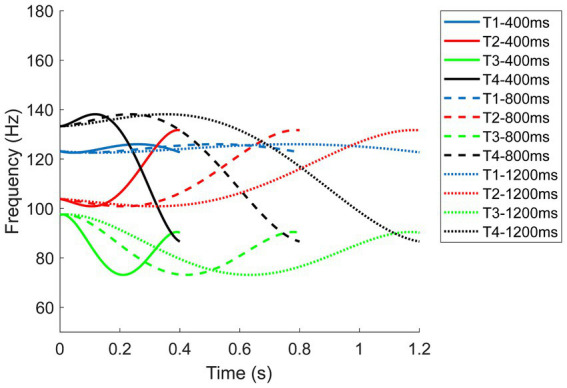
F0 contours of the four Mandarin tones at three duration levels for averaged male speakers.

In Xu’s (1997) research, the average durations of the four tones produced by eight native male speakers for the monosyllable *ma* were: Tone 1 at 247 ms, Tone 2 at 273 ms, Tone 3 at 349 ms, and Tone 4 at 214 ms. Even though tones vary intrinsically in duration, it is not a reliable distinguishing feature, especially when L2 learners are exposed to tone stimuli from various speakers in different contexts. To prevent L2 learners from using duration differences as a cue, we normalized the tones to ensure they all had the same duration. Besides, the initial duration level was 400 ms which is slightly longer than the average in Xu’s (1997) research to improve baseline performance. The duration was then further extended to 800 ms and 1,200 ms (see Figure 3) to assess whether exaggerated tones in duration improved tone identification accuracy.

#### Token synthesis

Finally, the four Mandarin tone patterns were superimposed onto the syllables in Praat (Boersma and Weenink, 2019; see Supplementary material for Praat code). The three Mandarin syllables used in this study were *ma*, *mo*, and *ya*. The syllable *ma* was chosen for its high familiarity to English speakers, as it resembles the basic phoneme for “mother” in the US and Canada. The syllables *mo* and *ya* were selected to control for syllable variations: *mo* has the same consonant but a different vowel as *ma*, while *ya* has a different consonant but the same vowel as *ma*. This selection ensures a controlled comparison while maintaining phonetic variety. In general, we had four acoustic factor variables: F0 expansion, duration, speaker sex, and syllable. This combination yielded a total of 216 stimuli (3 syllables × 4 tones × 3 durations × 3 F0 expansions × 2 speaker sexes).

### Procedure and learning paradigm

We manipulated tone as a within-subject variable while duration, F0 expansion, syllables, and speaker sexes were between-subject variables. Participants were randomly assigned to one of the four acoustic factor groups, each completing a total of 216 trials for training, testing, and generalization phases (see Table 1). The first 72 trials were for training and presented in random order. The subsequent 144 trials, which included a repetition of the 72 studied items for testing and 72 new stimuli for generalization, were also presented in random order. Therefore, participants were exposed to 144 stimuli which were just two-thirds of the total stimuli. This was done to reduce the number of total trials and minimize participant fatigue during the single session of training, testing, and generalization.

**Table 1:** Breakdown of the 24 learning conditions.

Factors	Conditions	Trials 1–72	Trials 73–216
Syllable	1	ma	ma & ya
	2	ma	ma & mo
	3	ya	ya & ma
	4	ya	ya & mo
	5	mo	mo & ma
	6	mo	mo & ya
Duration	7	400 ms	400 ms & 800 ms
	8	400 ms	400 ms & 1,200 ms
	9	800 ms	800 ms & 400 ms
	10	800 ms	800 ms & 1,200 ms
	11	1,200 ms	1,200 ms & 400 ms
	12	1,200 ms	1,200 ms & 800 ms
Expansion	13	1.0 expanded	1.0 expanded & 1.4 expanded
	14	1.0 expanded	1.0 expanded & 1.8 expanded
	15	1.4 expanded	1.4 expanded & 1.0 expanded
	16	1.4 expanded	1.4 expanded & 1.8 expanded
	17	1.8 expanded	1.8 expanded & 1.0 expanded
	18	1.8 expanded	1.8 expanded & 1.4 expanded
Speaker sex	19	male (ma, ya)	male (ma, ya) & female (ma, ya)
	20	female (ma, ya)	female (ma, ya) & male (ma, ya)
	21	male (ma, mo)	male (ma, mo) & female (ma, mo)
	22	female (ma, mo)	female (ma, mo) & male (ma, mo)
	23	male (ya, mo)	male (ya, mo) & female (ya, mo)
	24	female (ya, mo)	female (ya, mo) & male (ya, mo)

Since there were three levels for F0 expansion, duration, and syllable, it was straightforward to divide the stimuli based on these acoustic levels: one-third for training and testing, and another one-third for generalization. For participants in these three acoustic factor groups, the first 72 trials introduced one level of the acoustic factor. In the generalization phase, there were 72 new stimuli with a different acoustic level. The order of presentation for the acoustic levels was counterbalanced within each acoustic factor group (see Table 1 Conditions 1–18). For example, in Condition 7, participants began with tones of 400 ms (72 items = 4 tones × 3 F0 expansions × 3 syllables × 2 speaker sexes). The subsequent 144 trials comprised repetitions of the 400 ms trials and an additional 72 trials featuring tones of 800 ms. Regarding the speaker sex variable, which had only two levels (108 stimuli of male voice = 4 tones × 3 F0 expansions × 3 syllables × 3 durations, and 108 stimuli of female voice), we strategically used syllables to ensure participants were exposed to 144 stimuli, consistent with the other acoustic factor groups. Each participant experienced 2 out of the 3 syllables (144 stimuli = 2/3 of 216 stimuli) throughout the session. This included 72 stimuli with a male voice and 72 with a female voice. The usage of syllables was counterbalanced within the speaker sex group (see Table 1 Conditions 19–24).

**Figure 4:**
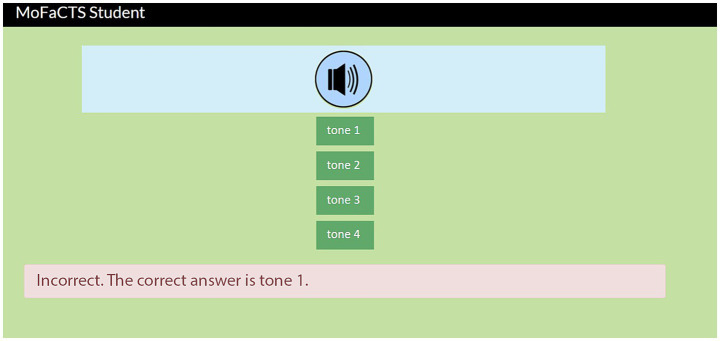
MoFaCTS interface for collecting web-based responses in the tone learning paradigm.

We used the Mobile Fact and Concept Training System (MoFaCTS) to train learners (see Figure 4). MoFaCTS is a multimedia flashcard learning system and educational research tool that can schedule practice for users, either “optimally” or for experimental conditions (Pavlik et al., 2016). In each trial, participants listened to a tone stimulus and selected its label from four tone options within 6 s. If they did not select an option within 6 s, their input was labeled as a “Timeout.” If they were correct, the system would provide feedback for 1 s, and immediately proceed to the next item. If they were incorrect, the system replayed the tone and displayed the correct answer, followed by a 6 s pause to allow participants to learn from the feedback.

Feedback was provided during all phases, including training, testing, and generalization. There were two main reasons for providing feedback during tests (testing and generalization). First, Mandarin tones are difficult for speakers of non-tonal languages (e.g., English) to acquire quickly. Previous research trained students enrolled in Elementary Chinese for a week and found only an 8.21% improvement in tone perception in the adaptive training group (Shih et al., 2010). The present study had only one session with 72 training trials, so performance improvement might be minimal with such limited training. Providing feedback during tests not only assessed participants’ performance but also enhanced it, leading to greater improvement. Second, we used high-variability training, and the tests included 144 trials, making for a long session. Feedback also served as a motivator to keep participants engaged. The correct feedback, lasting 1 s, acted as a bonus to expedite the task, while the incorrect feedback, lasting 6 s, served as a punishment, encouraging careful responses. Without feedback, participants might disengage, resort to random clicking, or become distracted until the session ends.

Finally, the dependent variable was the participant’s performance in the trials. For motivation, there was straightforward scoring on each trial (1 = correct, 0 = incorrect response including timeout responses).

## Results

### Learning and transfer performance

**Figure 5:**
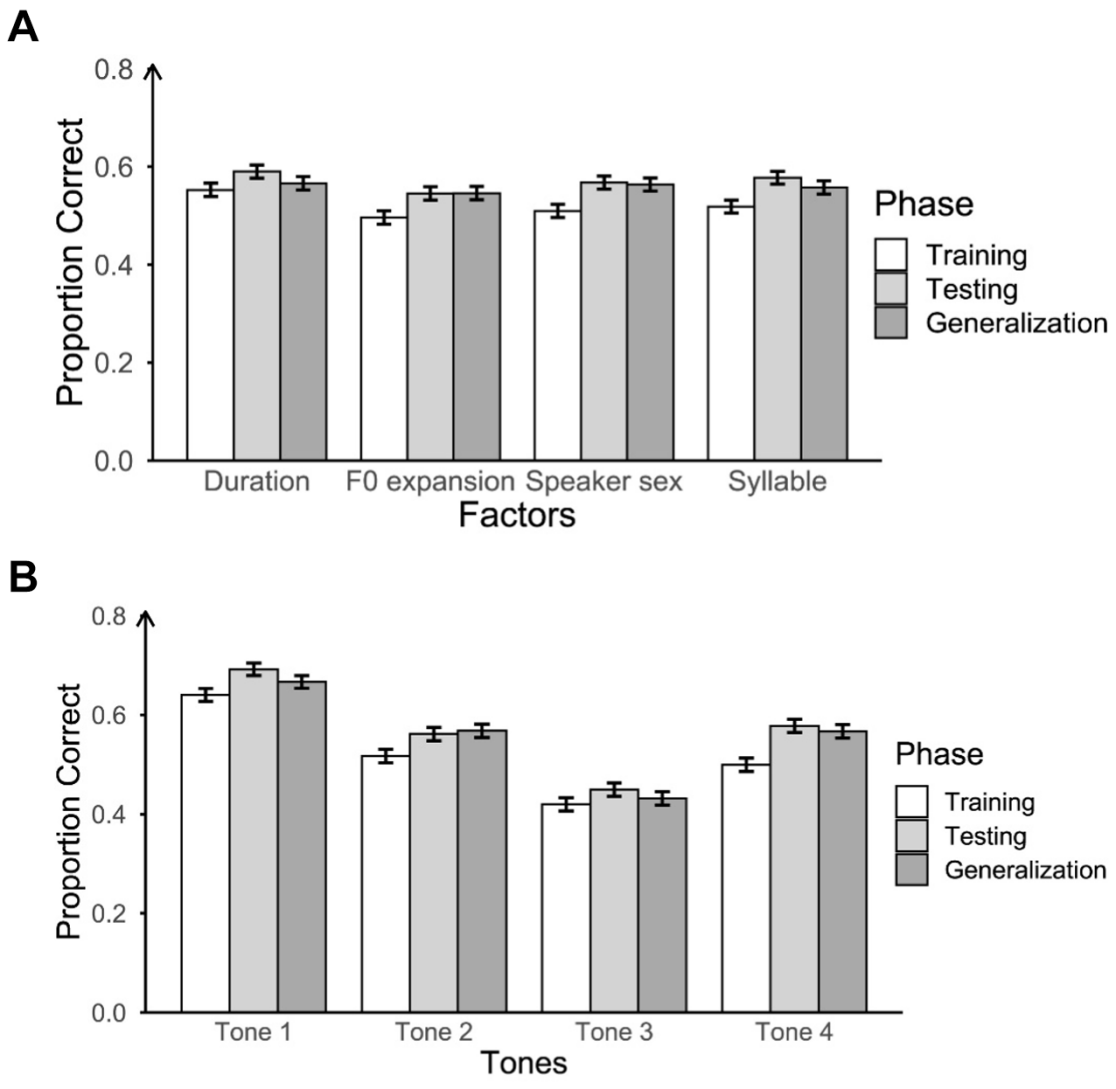
Proportion of correct responses for three phases across acoustic factors **(A)** and tones **(B)**, with error bars representing 95% confidence intervals. Chance-level performance in this task was 0.25.

We first assessed the average accuracy of participants in the three phases (training, testing, and generalization). This assessment was conducted for each acoustic factor group (speaker sex, duration, F0 expansion, and syllable; see Figure 5A), and for each tone (see Figure 5B). We considered the acoustic factors at a general level by averaging the scores of the sublevels within each acoustic factor to explore the differences among them in relation to phases and tones. For example, we averaged the three levels of duration (400 ms, 800 ms, and 1,200 ms) within the acoustic factor and compared it with the average scores of other acoustic factors.

To explore whether accuracy differences were observed in different phases across acoustic factors and tones, we used mixed-effects logistic regression models for the analyses. In the models, the fixed factors were the main effects of phases, acoustic factors, tones, and their interactions. Spearman correlations indicated no relation between age and average accuracy (*r* = 0.05, *p* = 0.39) but revealed a positive correlation between musical experience and average accuracy (*r* = 0.24, *p* < 0.001). Thus, we decided to include only music training experience in subsequent analyses. Given that 62.0% of participants had no formal music training, while a small proportion had varying levels of musical experience, we encoded musical experience as a categorical variable (0 years of music training vs. >0 years of music training) and treated it as a covariate. The dependent variable was participants’ accuracy on each trial (1, correct; 0, incorrect). Participant ID and item were considered as random factors. We used the *glmer* function from the package *lme4* (Bates et al., 2015) in the R environment (R Core Team, 2017) with the *nlminb* optimization method. Models and their comparison results were obtained using the *anova* function. The best fitting model should have the lowest Akaike Information Criterion (AIC, Akaike, 1998) and significant improvement of model fit in the Chi-square test (see Supplementary material for all model comparisons). Model comparisons indicated significant interactions between phases and tones, and between acoustic factors and tones. There was no interaction effect between phases and acoustic factors, nor was there a three-way interaction among acoustic factors, tones, and phases. Furthermore, the musical experience did modulate participants’ performance. The final model with the best model fit includes fixed effects of phases and tones, interactions between phases and tones and between acoustic factors and tones, music as a covariate, and random effects for participants and items.

We performed pairwise comparisons using estimated marginal means (with Bonferroni adjusted *p*-values) from the *emmeans* package in R (Lenth, 2018) to examine the main effects of phases, tones, and their interaction. For the interaction between phases and tones, pairwise comparisons among the three phases for each tone showed that accuracy in the testing phase was significantly higher than in the training phase across all tones (see Table 2). Accuracy in the generalization phase was also significantly higher than in the training phase except for Tone 3. This indicates that participants did not transfer well to the new stimuli for Tone 3. Furthermore, there was no significant difference in accuracy between the generalization and testing phases, except for Tone 1. Notably, the accuracy during the testing phase was significantly greater than that in the generalization phase for Tone 1, suggesting that participants’ ability to generalize Tone 1 was not as effective as their performance during the testing phase. For the main effect of phases, pairwise comparisons revealed that participants’ accuracy in both the testing phase and the generalization phase was significantly higher than that in the training phase. Even though there was no significant difference between the testing phase and the generalization phase on accuracy, the results indicated that participants learned through practice, and they transferred well to the new stimuli. For the main effect of tones, pairwise comparisons revealed that Tone 1 was the one that participants had the highest accuracy whereas Tone 3 was the one with lowest accuracy. There was no significant difference between Tone 2 and Tone 4 in accuracy. Thus, participants’ identification accuracy on each tone was in the following order: Tone 3 < (Tone 2 = Tone 4) < Tone 1.

**Table 2:** Pairwise comparisons for the effects of phase and tone.

Pairwise comparisons	Estimate	Std.Error	*z*	*p*-value
**Phases**				
Testing - Training	0.27	0.02	11.92	<0.0001
Generalization - Training	0.22	0.02	9.78	<0.0001
Generalization - Testing	−0.05	0.02	−2.11	0.104
**Tones**				
Tone 1 - Tone 2	0.62	0.13	4.89	<0.0001
Tone 1 - Tone 3	1.23	0.13	9.70	<0.0001
Tone 1 - Tone 4	0.63	0.13	4.96	<0.0001
Tone 2 - Tone 3	0.61	0.13	4.82	<0.0001
Tone 2 - Tone 4	0.01	0.13	0.07	1.000
Tone 3 - Tone 4	−0.60	0.13	−4.75	<0.0001
**Tone 1**				
Testing - Training	0.28	0.05	6.22	<0.0001
Generalization - Training	0.14	0.05	3.16	0.005
Generalization - Testing	−0.14	0.05	−3.04	0.007
**Tone 2**				
Testing - Training	0.22	0.04	5.02	<0.0001
Generalization - Training	0.26	0.04	5.95	<0.0001
Generalization - Testing	0.04	0.04	0.95	1.000
**Tone 3**				
Testing - Training	0.16	0.05	3.48	0.002
Generalization - Training	0.10	0.05	2.12	0.103
Generalization - Testing	−0.06	0.05	−1.36	0.520
**Tone 4**				
Testing - Training	0.41	0.04	9.11	<0.0001
Generalization - Training	0.38	0.04	8.47	<0.0001
Generalization - Testing	−0.03	0.04	−0.64	1.000

Glmer [correct ~ Phase * Tone + Factor: Tone + music + (1| subject) + (1|item), family = “binomial”]. The *p* values are Bonferroni adjusted.

Regarding the significant interaction between acoustic factors and tones, pairwise comparisons among tones for each acoustic factor, with Bonferroni-adjusted *p*-values, indicated a consistent pattern in tone identification performance (see Supplementary Table S2). The performance rankings within each acoustic factor group were the same as noted above: Tone 3 < (Tone 2 = Tone 4) < Tone 1. Additionally, pairwise comparisons among acoustic factors for each tone did not reveal significant differences in tone identification performance (see Supplementary Table S2). For instance, participants’ average performance in the duration group did not differ from that in the F0 expansion group, speaker sex group, or syllable group across all four tones.

To further pinpoint whether specific modifications of acoustic factors, such as F0 expansion (1.0 expanded, 1.4 expanded, and 1.8 expanded), would facilitate Mandarin tone learning for English speakers, we conducted additional analyses to investigate how modifying each acoustic factor influenced participants’ tone identification accuracy, as shown in the following sections.

### Effects of F0 expansion on tone identification accuracy

**Figure 6:**
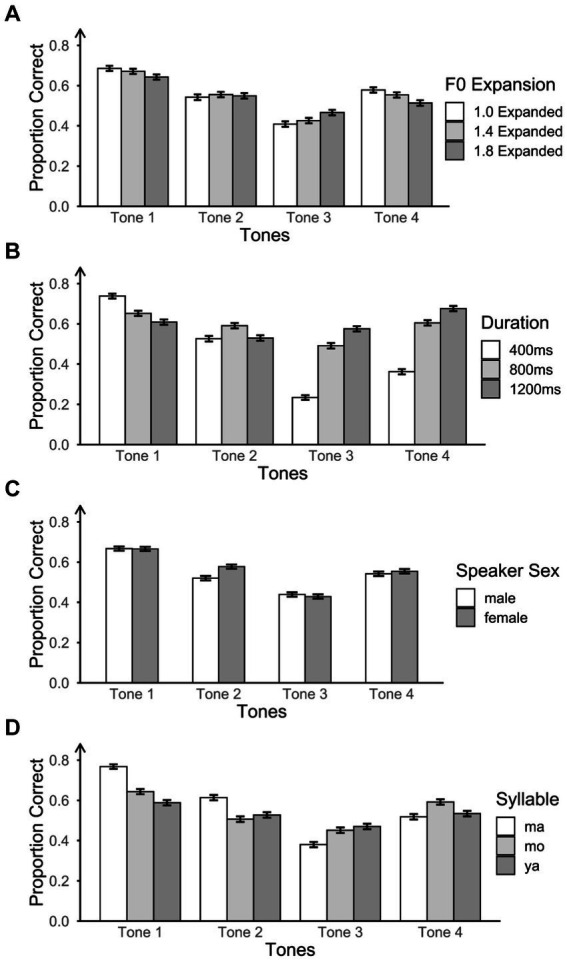
Proportion of correct responses for four tones across each acoustic factor: **(A)** three F0 expansion levels, **(B)** three durations, **(C)** two speaker sexes, and **(D)** three syllables.

Figure 6A shows participants’ accuracy at three F0 expansion levels by four tones. We also conducted mixed effects logistic regression model analyses to explore whether expanded F0 in frequency promotes tone identification across the four tones. The term *Items* was removed from the random effects in the following analyses since it is highly correlated with the fixed effects (e.g., F0 expansion and tone) which may induce collinearity between fixed and random effects. Therefore, we included only one random effect: participants. Model comparisons revealed that the model with the best model fit is the one with F0 expansion, tone, and their interaction as the fixed effect, music as the covariate, and participants as the random effect.

The pairwise comparison results are shown in Table 3. Generally, participants performed better on moderate F0 expansion (1.4) compared to lower (1.0) and higher (1.8) F0 expansion. The effect of F0 expansion on tone identification varied across the four tones. For Tone 1, participants exhibited significantly higher accuracy on expansion 1.0 and expansion 1.4 compared to expansion 1.8. However, there was no statistically significant difference in performance between expansion 1.0 and 1.4. Therefore, on Tone 1, the order of participants’ accuracy can be summarized as follows: expansion 1.0 = expansion 1.4 > expansion 1.8. In contrast, for Tone 2, participants’ accuracy on expansion 1.0 was significantly lower than on expansion 1.4 and expansion 1.8, with no significant difference between expansion 1.4 and 1.8. Consequently, the order of accuracy for Tone 2 was as follows: expansion 1.0 < expansion 1.4 = expansion 1.8. For Tone 3, the order of accuracy was observed as expansion 1.0 < expansion 1.4 < expansion 1.8. For Tone 4, the accuracy order was the same as for Tone 1, with expansion 1.0 = expansion 1.4 > expansion 1.8. Collectively, these results suggest that larger F0 expansion promotes tone identification for Tone 2 and Tone 3 but not for Tone 1 and Tone 4.

**Table 3:** Pairwise comparisons for the effects of F0 expansion and tone.

Pairwise comparisons	Estimate	Std.Error	*z*	*p*-value
**Expansion**				
1.4 expanded- 1.0 expanded	0.08	0.02	3.43	0.002
1.8 expanded - 1.0 expanded	0.03	0.02	1.38	0.507
1.8 expanded - expansion1.4	−0.05	0.02	−2.10	0.106
**Tone 1**				
1.4 expanded- 1.0 expanded	0.02	0.05	0.34	1.000
1.8 expanded - 1.0 expanded	−0.13	0.05	−2.92	0.010
1.8 expanded - expansion1.4	−0.15	0.04	−3.32	0.003
**Tone 2**				
1.4 expanded- 1.0 expanded	0.15	0.04	3.56	0.001
1.8 expanded - 1.0 expanded	0.12	0.04	2.79	0.016
1.8 expanded - expansion1.4	−0.03	0.04	−0.77	1.000
**Tone 3**				
1.4 expanded- 1.0 expanded	0.17	0.04	3.90	<0.001
1.8 expanded - 1.0 expanded	0.36	0.04	8.19	<0.0001
1.8 expanded - expansion1.4	0.19	0.04	4.33	<0.0001
**Tone 4**				
1.4 expanded- 1.0 expanded	−0.03	0.04	−0.64	1.000
1.8 expanded - 1.0 expanded	−0.22	0.04	−5.11	<0.0001
1.8 expanded - expansion1.4	−0.19	0.04	−4.52	<0.0001

Glmer[correct ~ Expansion * Tone + music + (1| subject), family = “binomial”]. The *p* values are Bonferroni adjusted.

**Figure 7:**
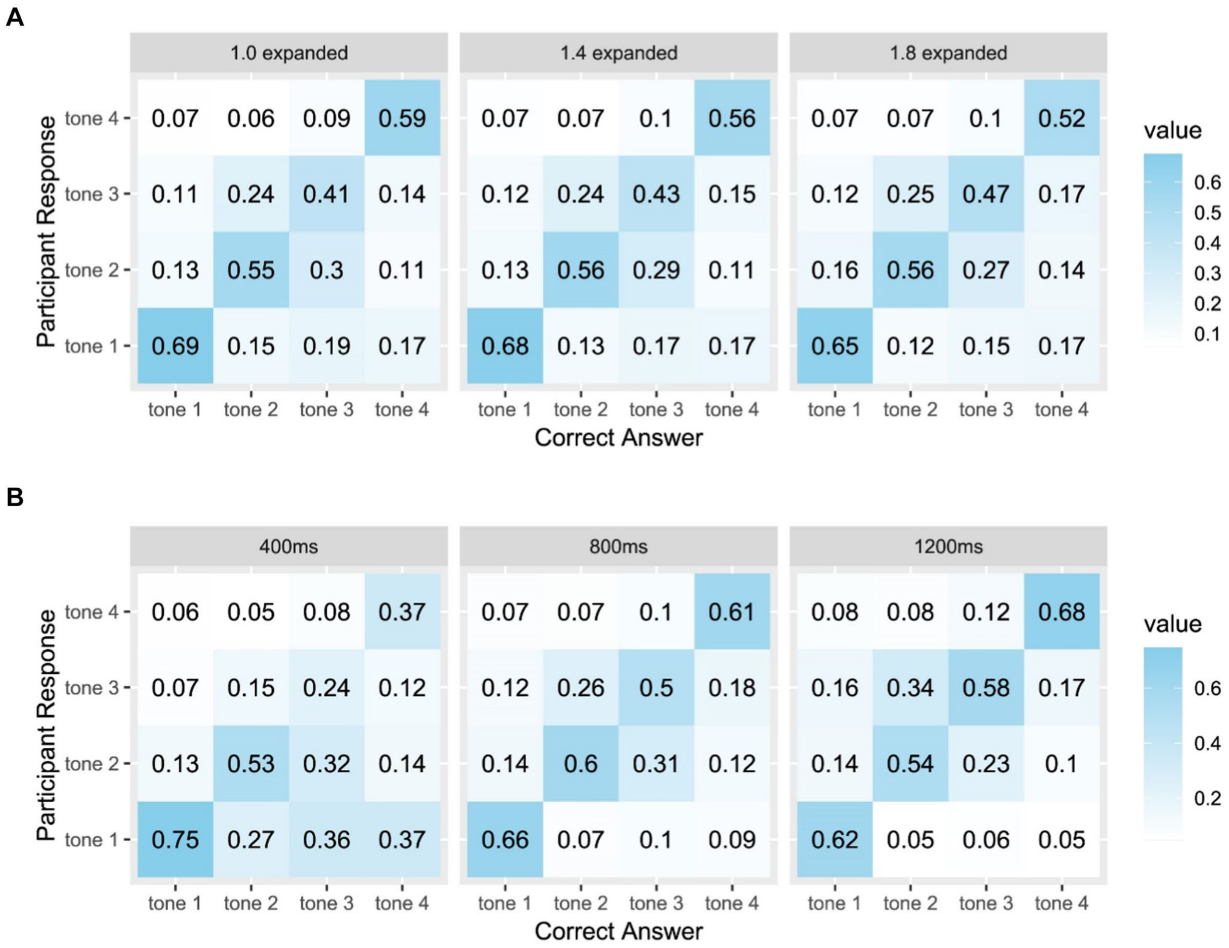
Tone confusion matrices **(A)** for each F0 expansion level and **(B)** for each duration. Columns correspond to the correct tone category, rows to the participants’ responses. Values are the relative frequency (proportion) of participants’ responses for each correct category.

To deepen our understanding of how varying levels of F0 expansion impact tone perception, we analyzed the tonal confusion matrices for each F0 expansion level across tones, excluding participants’ timeout responses to focus solely on instances of confusion between the correct answer and other options. The results showed similar findings as the mixed regression model analysis (see Figure 7A). With increasing F0 expansion, participants exhibit a higher proportion of correct responses for Tone 3, rising from 0.41 to 0.47. Additionally, the proportion of incorrect responses for Tone 2, when the correct answer was Tone 3, decreased from 0.3 to 0.27. Although the effect of F0 expansion on Tone 2 was less marked compared to Tone 3, there was a slight increase in the accuracy of responses for Tone 2, indicating that expanded F0 does contribute to improved tone perception of Tone 2 and Tone 3. Contrastingly, the accuracy in identifying Tone 1 and Tone 4 showed a downward trajectory with increased F0 expansion.

### Effects of duration on tone identification accuracy

Figure 6B summarizes the proportion of correct responses at three duration levels by four tones. We conducted mixed-effects logistic regression to investigate whether extended duration positively influences tone identification across the four tones. Model comparisons revealed that the model with the best model fit was the one containing duration, tone, and their interaction as the fixed effect, music as the covariate, and participants as the random effect.

We found the main effects of tone and duration, as well as their interaction. Given that the pairwise comparisons of tones have been reported in the previous section, our focus here was solely on pairwise comparisons for the three duration levels, both at a general level and for each specific tone (see Table 4). In general, participants’ identification accuracy at 400 ms was significantly lower than that at 800 ms and 1,200 ms. There was no significant difference between the 800 ms and 1,200 ms in accuracy. However, the effect of duration on accuracy varied across tones. For Tone 1, participants’ accuracy at 400 ms was significantly higher than at 800 ms and 1,200 ms. Participants also performed significantly better on 800 ms than on 1,200 ms. Thus, on Tone 1, the order of participants’ accuracy was 400 ms > 800 ms > 1,200 ms. However, on Tone 2, participants had significantly higher accuracy at 800 ms than at 400 ms and 1,200 ms but had no difference between the 400 ms and 1,200 ms (400 ms = 1,200 ms < 800 ms). Furthermore, Tone 3 and Tone 4 had the opposite order of accuracy (1,200 ms > 800 ms > 400 ms) as to Tone 1. This suggests that participants achieved better performance on longer duration stimuli for Tone 3 and 4 but worse performance on Tone 1, where shorter tokens were more conducive to identification.

**Table 4:** Pairwise comparisons for the effects of duration and tone.

Pairwise comparisons	Estimate	Std.Error	*z*	*p*-value
**Phase**				
800 ms-400 ms	0.63	0.02	26.76	<0.0001
1,200 ms-400 ms	0.67	0.02	28.55	<0.0001
1,200 ms-800 ms	0.04	0.02	1.72	0.257
**Tone 1**				
800 ms-400 ms	−0.43	0.05	−9.34	<0.0001
1,200 ms-400 ms	−0.67	0.05	−14.68	<0.0001
1,200 ms-800 ms	−0.24	0.04	−5.44	<0.0001
**Tone 2**				
800 ms-400 ms	0.35	0.04	7.97	<0.0001
1,200 ms-400 ms	0.03	0.04	0.58	1.000
1,200 ms-800 ms	−0.32	0.04	−7.40	<0.0001
**Tone 3**				
800 ms-400 ms	1.39	0.05	29.39	<0.0001
1,200 ms-400 ms	1.77	0.05	37.38	<0.0001
1,200 ms-800 ms	0.38	0.04	8.83	<0.0001
**Tone 4**				
800 ms-400 ms	1.21	0.04	27.17	<0.0001
1,200 ms-400 ms	1.55	0.05	34.11	<0.0001
1,200 ms-800 ms	0.34	0.04	7.52	<0.0001

Glmer [correct ~ Duration * Tone + music + (1| subject), family = “binomial”]. The *p* values are Bonferroni adjusted.

We further examined the tonal confusion matrices for each duration across tones (see Figure 7B). The analysis revealed that participants made more errors when presented with longer durations of Tone 1. Conversely, increasing the duration led to a higher proportion of correct responses for Tone 3 and Tone 4. As the confusion matrices corroborated the results obtained from the mixed regression model analyses, we opted not to include additional confusion matrices in subsequent analyses due to limitations in paper length.

### Effects of speaker sex on tone identification accuracy

Figure 6C shows participants’ accuracy as a function of speaker sex across the tones. Again, we used mixed effects logistic regression models to explore whether the higher F0/female voice positively influences tone identification compared to male voice. Model comparisons revealed that the best-fitting model included the main effects of speaker sex and tone, their interaction as the fixed effect, music as the covariate, and participants as the random effect.

Pairwise comparisons are shown in Table 5. Participants’ accuracy was higher for the female compared to the male speaker. However, the effect of speaker sex on tone identification varied across tones. For Tone 1 and Tone 4, there was no sex difference in participants’ accuracy. For Tone 2, performance was higher for the female voice compared to male voice. For Tone 3, the male voice had higher accuracy than the female voice.

**Table 5:** Pairwise comparisons for the effects of speaker sex and tone.

Pairwise comparisons	Estimate	Std.Error	*z*	*p*-value
**Speaker sex**				
Male - female	−0.05	0.02	−2.67	0.008
**Tone 1**				
Male - female	0.03	0.04	0.74	0.458
**Tone 2**				
Male - female	−0.25	0.03	−7.19	<0.0001
**Tone 3**				
Male - female	0.07	0.04	2.02	0.043
**Tone 4**				
Male - female	−0.04	0.03	−1.08	0.281

Glmer [correct ~ Speaker sex * Tone + music + (1| subject), family = “binomial”]. The *p* values are Bonferroni adjusted.

### Effects of syllable on tone identification accuracy

Figure 6D shows participants’ accuracy at three syllable levels by four tones. We also performed mixed-effects logistic regression model analyses to investigate potential variations in tone identification accuracy among different syllables. Model comparisons revealed that the model with the best model fit was the one with syllable, tone, and their interaction as the fixed effect, music as the covariate, and participants as the random effect.

Pairwise comparisons are shown in Table 6. Generally, participants’ accuracy on syllable *ma* was higher than syllable *mo* and *ya*. Furthermore, syllable *mo* also had a significantly higher accuracy than syllable *ya*, resulting in the overall order of accuracy being *ma* > *mo* > *ya*. However, the effect of syllables on tone identification varied across four tones. For Tone 1, participants’ accuracy on the three syllables followed the same order as the general situation (*ma* > *mo* > *ya*). For Tone 2, the order of participants’ accuracy was *ma* > *mo* = *ya*. However, for Tone 3, the order was the opposite of Tone 2 (*ma* < *mo* = *ya*). Lastly, for Tone 4, the order was *mo* > *ma* = *ya*. Thus, syllable *ma* resulted in better performance for Tone 1 and Tone 2 but worse performance for Tone 3 and Tone 4.

**Table 6:** Pairwise comparisons for the effects of syllable and tone.

Pairwise comparisons	Estimate	Std.Error	*z*	*p*-value
**Session**				
mo - ma	−0.16	0.02	−6.67	<0.0001
ya - ma	−0.31	0.02	−13.14	<0.0001
ya – mo	−0.15	0.02	−6.64	<0.0001
**Tone 1**				
mo - ma	−0.73	0.05	−15.32	<0.0001
ya - ma	−1.05	0.05	−22.33	<0.0001
ya – mo	−0.33	0.04	−7.41	<0.0001
**Tone 2**				
mo - ma	−0.55	0.04	−12.45	<0.0001
ya - ma	−0.51	0.04	−11.68	<0.0001
ya – mo	0.03	0.04	0.72	1.00
**Tone 3**				
mo - ma	0.32	0.04	7.34	<0.0001
ya - ma	0.34	0.04	7.75	<0.0001
ya – mo	0.02	0.04	0.42	1.00
**Tone 4**				
mo - ma	0.32	0.04	7.29	<0.0001
ya - ma	−0.02	0.04	−0.44	1.00
ya – mo	−0.34	0.04	−7.73	<0.0001

Glmer [correct ~ Syllable * Tone + music + (1| subject), family = “binomial”]. The *p* values are Bonferroni adjusted.

## Discussion

In this study, we sought to understand the intricate challenges faced by L2 learners in lexical tone perception, particularly focusing on the impact of exaggerated acoustic properties on facilitating Mandarin tone learning for English speakers. Based on the findings from tone hyperarticulation research, our study implemented three key modifications: expanding the F0 range, increasing the F0, and lengthening the overall duration. To precisely manipulate the pitch contours, we systematically applied a synthetic method during the creation of the tone stimuli. Our key goals were to assess the impact of F0 expansion, F0 height, duration, and varied syllables on the complexity of Mandarin tone learning and generalization.

We found participants’ musical experience played a modulating role in their tone perception performance. This aligns with prior research indicating that musical training experience enhances tone perception for English musicians (Schön et al., 2004; Alexander et al., 2005; Marques et al., 2007; Wong et al., 2007; Chandrasekaran et al., 2009; Bidelman et al., 2011, 2013; Zhao and Kuhl, 2015). Although there were no differences among acoustic factors during the three phases (training, testing, and generalization), participants demonstrated improvements in accuracy from training to testing and later generalization. This suggests the effectiveness of perceptual training in enhancing Mandarin tone learning for adult English speakers, aligning with established research findings (Wang et al., 1999; Francis et al., 2008; Shih et al., 2010; Liu et al., 2011; Zhao and Kuhl, 2015; Shen and Froud, 2016; Reetzke et al., 2018).

Notably, participants encountered challenges in generalizing Tone 3, indicating difficulties in transferring learned patterns to new stimuli for this tone. This difficulty aligns with previous observations that Tone 3 poses challenges for both native and non-native speakers (Yue-Hashimoto, 1986). Our study identified Tone 1 as the easiest to perceive, whereas Tone 3 emerged as the most challenging. These findings support established hierarchies of tonal acquisition difficulty for native Chinese children (Li and Thompson, 1977) and native English speakers (Yue-Hashimoto, 1986). It is noteworthy that different studies have yielded varying results with regard to the difficulty of Mandarin tone learning in diverse learning contexts. For example, Lee et al. (2010) found Tone 2 to be the most difficult to perceive for college students who were taking Mandarin classes, and Broselow et al. (1987) identified Tone 4 as the easiest for English adult speakers to identify when presented in isolation. The possible reason for the discrepancy in tone perception difficulty may lie in the use of different stimuli. For example, Lee et al. (2010) and Broselow et al. (1987) utilized natural stimuli produced by native Mandarin speakers without controlling their acoustic features, such as duration. Tone 3 often exhibits a longer duration than Tone 2, implying that duration serves as a perceptually relevant acoustic cue for tone distinction (Blicher et al., 1990). However, our study used synthesized tones with normalized durations, limiting participants’ use of duration as a distinguishing cue.

Analyzing the influence of each acoustic factor (duration, F0 expansion, speaker sex, and syllable) on tone identification accuracy, our findings indicate that the impact of these factors on tone identification is tone-specific, emphasizing the need for nuanced considerations in acoustic factor selection during training. The results deviated from the straightforward expectation that a simple expansion of pitch contours would enhance tone learning, as there was a significant interaction between F0 expansion and tones. Notably, for Tone 2 and 3, heightened F0 expansion corresponded to improved performance, partially aligning with our initial hypothesis. However, for Tone 1 and Tone 4, the most expanded F0 resulted in the poorest performance.

The beneficial effect of expanded F0 on the perception of Tone 2 and 3 can be attributed to their distinct pitch contours. Tone 2, characterized by a rising pitch, and Tone 3, featuring a low dipping pitch, have been shown in previous research to rely on the timing of the F0 turning point as a crucial perceptual cue for differentiation (Shen and Lin, 1991; Moore and Jongman, 1997; Wang et al., 1999). The F0 contours of Tone 2 and Tone 3 reveal that the turning point occurs earlier and more gradually for Tone 2 compared to Tone 3 (see Figure 2). Expanding the F0 of Tone 3 results in a stretching of the turning point, making it substantially lower than in the non-expanded version of Tone 3. This adjustment may enhance the distinctiveness of Tone 3, particularly in contrast to Tone 2. The examination of tonal confusion matrices further supports this speculation (see Figure 7A). Although there was a high confusion between Tone 2 and Tone 3, consistent with previous research (Wang et al., 1999), increasing F0 expansion can enhance the perception and discrimination of Tones 2 and 3. However, for Tone 1 and Tone 4, which feature a level and a falling pitch, respectively, and do not rely on turning points for discrimination, expanded F0 may introduce additional curvature, potentially leading to confusion with other tones.

Similarly, longer durations did not universally improve the perception of all tones. For Tone 1, shorter durations yielded better performance among participants, whereas, for Tone 2, a medium duration was optimal. Conversely, for Tone 3 and Tone 4, longer durations led to better performance. These findings are primarily influenced by the distinct pitch contours exhibited by each tone. In natural speech, the duration differences among Mandarin tones are relatively small, typically within 10%. However, on monosyllabic or final syllables, Tone 3 tends to have a much longer duration compared to other tones, as it can even be split into two syllables to emphasize the turning point (Duanmu, 2007). This emphasis on the turning point may explain why participants exhibited better performance for Tone 3 with longer durations. Previous research suggested that longer durations might increase the detectability of the initial non-rising portion of the F0 contour for Tone 3, enhancing F0 cues when distinguishing Tone 3 from Tone 2 (Blicher et al., 1990). Duration may have a more significant effect on the curvilinear tones because participants need to monitor the entire pitch contour to perceive the variations in the F0 direction—whether it rises (Tone 2), falls (Tone 4), or follows both directions (Tone 3). For Tone 1, which ideally maintains an invariant F0, participants could recognize it after hearing approximately 200 ms of the F0. This shorter duration in perception integration for Tone 1 aligns with findings that Tone 1 has a relatively shorter duration when produced in isolation or in a phrase-final position (Blicher et al., 1990; Xu, 1997; Chen et al., 2017). However, the polynomial equations we used in our study were based on the productions of native Mandarin speakers, resulting in a slightly curvilinear representation of Tone 1. This curvature might not be noticeable in shorter durations but could be exaggerated in prolonged durations, making Tone 1 more confusable with other tones (see Figure 3). This was also suggested by the confusion matrices for different durations (see Figure 7B).

Considering speaker sex, a higher fundamental frequency (F0) in the female voice was advantageous for the identification of Tone 2 but had the opposite effect for Tone 3. This may be attributed to differences in the pitch contour and overall F0 of the tones. Tone 2 typically exhibits a rising pitch contour, where the F0 increases gradually over time. A higher F0 in the female voice could amplify this rising pattern, enhancing its perceptual salience and facilitating easier identification. Conversely, Tone 3, in addition to its pitch direction, features the lowest F0 among the four tones. A higher F0 in the female voice might diminish the salience of this characteristic, potentially making it more challenging to identify accurately. It is worth noting that the F0 of the female voice is typically twice that of the male voice, corresponding to an octave difference. Some researchers have suggested that the wider F0 range of female speakers contributes to their overall higher intelligibility (Bradlow et al., 1996). However, studies on the intelligibility of speaker sex have yielded inconsistent findings (Ferguson, 2004; Markham and Hazan, 2004; McCloy et al., 2015; Yoho et al., 2019). Individual differences in perceptual sensitivity to pitch changes across different F0 ranges may also play a role in the effect of speaker sex on tone perception. Further research is needed to delve into these potential explanations more comprehensively.

Although syllable manipulation was not the primary focus of our study, we observed a significant interaction between tone and syllable. Specifically, participants exhibited better performance on the syllable *ma* for Tone 1 and Tone 2, while for Tone 3 and 4, *ma* resulted in worse performance. All four Mandarin tones have a consistent alignment with the syllable regardless of internal syllable structure (Xu, 1998; Liu et al., 2011). In our study, consonant-vowel syllables served as carriers for tonal stimuli. The syllable *ma* is the basic phoneme for the word “mother” in the US and Canada, potentially facilitating participants’ connections of Tone 1 and Tone 2 to their native English language experiences as intonations (Francis et al., 2008). However, for Tone 3 and Tone 4, the worse performance of syllable *ma* may be due to the mismatch between English intonational patterns and Mandarin tone categories. Moreover, changing either the consonant or the vowel can affect tone perception. For instance, even though the syllable *ya* shares the same vowel as *ma*, it begins with the phoneme /j/, introducing a different acoustic dynamic. This dynamic may help participants focus more on the tone’s acoustic features, particularly for Tone 3, making it easier to perceive than the Tone 3 carried by syllable *ma*. The finding highlights the importance of syllable selection in tone perception studies. Future research should further explore the impact of different syllables on tone perception to better understand the nuances of tonal recognition and its implications for language learning and phonetic research.

In summary, our findings underscore the significant variability in perceived tone difficulty, influenced by acoustic factors including duration, pitch expansion, syllable, and speaker sex. This suggests the perceptual hierarchy observed in prior research is perhaps oversimplified, as we find tone difficulty varies across different acoustic dimensions. Our study contributes valuable insights into the complex interplay of acoustic factors in tone learning for English speakers. These findings hold considerable implications for the design of training programs tailored to accommodate the specific acoustic characteristics of each tone. Previous research underscores the importance of maintaining an optimal level of difficulty in enhancing training effectiveness (Kelley, 1969), suggesting that instructors could manipulate different acoustic properties to generate stimuli with varied difficulty levels and implement adaptive training approaches. By allowing beginners to start with easy tone stimuli and progressively introducing more challenging ones as their performance improves, the efficacy of L2 tone acquisition could be enhanced (Shih et al., 2010). Furthermore, using tone stimuli with diverse acoustic properties may be advantageous as it increases stimulus variability, potentially aiding learners in constructing a robust classification system. This approach aligns with theories of memory that underscore the significance of varied encoding experiences during the learning process (Lively et al., 1993; Shih et al., 2010; Reetzke et al., 2018).

One limitation of our study is the relatively small sample size for each condition (24 learning conditions), which restricted our ability to thoroughly investigate how ordering the presentation of our different acoustic factors would impact tone perception. For instance, we are unable to determine whether participants benefit more from starting with longer or shorter duration tones. Additionally, as the order of tones was not manipulated, we could not explore how the perception of one tone influences the perception of others. Future research could explore optimal sequencing of practice, such as progressing from easy to difficult tones (e.g., Tone 1—Tone 2—Tone 4—Tone 3) or from longer to shorter durations (e.g., 1,200 ms—800 ms—400 ms). Moreover, given the interactions between tones and acoustic factors, future studies may consider how sequencing in a learning task could account for these interactions.

## Conclusion

This study examined lexical tone learning using synthetic Mandarin tones, providing control over acoustic properties such as duration, F0 expansion, syllable, and speaker sex. Participants demonstrated improved performance through practice and successfully transferred their learning to new stimuli. Importantly, we observed that extended duration, expanded F0, and higher F0 (female voice) can facilitate the perception of certain tones. Even though these modifications of acoustic properties did not uniformly improve learning for all tones, instructors can still manipulate various acoustic factors to generate stimuli with diverse difficulty levels and select stimuli with suitable difficulty levels for L2 learners to facilitate their second language learning.

## Data Availability

The datasets analyzed for this study can be found at: https://osf.io/shjf4/files/osfstorage/65f860f618cee302bf1a2965. Further inquiries can be directed to the MC.
