# Supplementary material for: Enhancing lexical tone learning for second language speakers: effects of acoustic properties in Mandarin tone perception
Source: Front Psychol. 2024 Aug 21;15:1403816. doi: 10.3389/fpsyg.2024.1403816 (PMC11371754; doi:10.3389/fpsyg.2024.1403816)
Supplement: Supplementary file 1 [file Table_1.DOCX]

Supplementary Material

# MATLAB Code

clear all; close all; clc

fs=10000;

%----specify the parameters----------------

durs=[400:800:1200]/1000; %duration (sec)

%------------------------------------------

for kk=1:length(durs)

dur =durs(kk);

x=linspace(0,dur,fix(dur*fs))';

%---Create chinese tones------------------

%freq2semi(97.45,133.26); % # semitones of pitch excursion between tones

semishift=[0 0 0 0]; pitchType='close';

%semishift=[0 -1.75 -2.25 4.5]; pitchType='expanded';

T1 =semi2freq(123.16,semishift(1)) - (11.61/dur).*x + (67.94/(dur^2)).*x.^2 - (85.18/(dur^3)).*x.^3 + (28.43/(dur^4)).*x.^4;

T1m = mean(T1);

T1a = (T1-T1m)*1+T1m;

T1b = (T1-T1m)*1.4+T1m;

T1c = (T1-T1m)*1.8+T1m;

%T1f= 2*(T1-T1m)*1+2*T1m;

T2 =semi2freq(103.85,semishift(2)) - (8.45/dur).*x - (76.32/(dur^2)).*x.^2 + (297.91/(dur^3)).*x.^3 - (185.34/(dur^4)).*x.^4;

T2m = mean(T2);

T2a = (T2-T2m)*1+T2m;

T2b = (T2-T2m)*1.4+T2m;

T2c = (T2-T2m)*1.8+T2m;

%T2f= 2*(T2-T2m)*1+2*T2m;

T3 =semi2freq(97.45,semishift(3)) + (15.05/dur).*x - (430/(dur^2)).*x.^2 + (805/(dur^3)).*x.^3 - (397.27/(dur^4)).*x.^4;

T3m = mean(T3);

T3a = (T3-T3m)*1+T3m;

T3b = (T3-T3m)*1.4+T3m;

T3c = (T3-T3m)*1.8+T3m;

%T3f= 2*(T3-T3m)*1+2*T3m;

T4 =semi2freq(133.26,semishift(4)) + (4.36/dur).*x + (165.15/(dur^2)).*x.^2 - (504.84/(dur^3)).*x.^3 + (288.71/(dur^4)).*x.^4;

T4m = mean(T4);

T4a = (T4-T4m)*1+T4m;

T4b = (T4-T4m)*1.4+T4m;

T4c = (T4-T4m)*1.8+T4m;

%T4f= 2*(T4-T4m)*1+2*T4m;

stim=[T1'; T2'; T3'; T4']; %all stim pitch contours

disp(['Mean pitch (Hz): ' num2str(mean(mean(stim)))]) %average pitch height of all contours

pitchType='';

%------------------------------------------

%----Plot Chinese tones---------------------

figure; plot(x,stim(1,:),'r','LineWidth',2);axis([0 1 50 280])

hold on

plot(x,stim(2,:),'--r','LineWidth',2);

%title(['Chinese contours: ' num2str(1000*dur) ' ms, ' pitchType ' pitch'],'FontSize',18);

set(gca,'FontSize',14);

xlabel('Time','FontSize',14);

ylabel('Frequency','FontSize',14);

legend({'T1','T2','T3','T4'},'FontSize',14) ;

xlim([0 1.5]);drawnow

%-------------------------------------------

tt=linspace(0,dur,dur*fs);

for j=1:size(stim,1)

% tempPitch=interp1(x,stim(j,:),tt);

% tempPitch=[stim(j,:) stim(j,end)*ones(1,0.100*fs)];

tempPitch=stim(j,:);

tempPitch(1)=tempPitch(2);

sig=makedIRN(tempPitch,fs,128,1,[50 1000]); %generate pitch contour with IRN

%sig(j,:) =pitchSweep(tempPitch,fs); %complex tone

rmsx=sqrt(mean(sig.^2));

sig=rampstim((0.15/rmsx)*sig',10,fs); %match RMS to same value

audiowrite(['T' num2str(j) '_' num2str(1000*dur) 'ms_' pitchType '.wav'],sig,fs);

end

%sound(sig(4,:), fs); %play

print('-dtiff',['tones_' num2str(1000*dur) 'ms_' pitchType '.tiff']); %print figure

clear sig stim tempPitch T1 T2 T3 T4;

end

%run extractPitch_synth_hum.praat to overlay on vowel sound

# Praat Code

# This program reads in .WAV file from the specified directory and

# outputs a new WAV file with the same pitch contour but synthesized

#

# Program written by: Gavin Bidelman,PhD <gbidel@iu.edu> Oct 2017

#---Prompt user for directory where .WAV files are located-----

form Batch WAV replace pitch onto vowel

sentence input_directory D:\old laptop\F\desktop files\new tone\Male\hum

#---this value is the new sampling frequency-----

integer new_Fs 44100

integer precision 50

word vowel ma

endform

#read vowel and create manipulation object

#fileName1$="ma_model.wav"

#Read from file... 'input_directory$'\'fileName1$'

Create Strings as file list... fileList 'input_directory$'\*.wav

#---The name of files -----

numberOfFiles = Get number of strings

for i to numberOfFiles

select Strings fileList

#---finding the name of each file-----

fileName$ = Get string... i

Read from file... 'input_directory$'\'fileName$'

#select Sound

dur1=Get total duration

#writeInfoLine: string$(dur1)

Resample... 'new_Fs' 'precision'

To Pitch: 0, 40, 750

Down to PitchTier

#----creae vowel of same duration

Create SpeechSynthesizer: "cmn", "default"

#compute "speech rate for synthesizer (words per sec) from duration of stim

#newDur=dur*60

Set speech output settings: 'new_Fs',0.01, 50, 50, 50, "yes", "IPA"

To Sound: vowel$, "no"

Rename: fileName$

dur2=Get total duration

durRatio=dur1/dur2

Lengthen (overlap-add): 40, 750, 'durRatio'

To Manipulation: 0.01, 40, 750

Rename: fileName$

name$ = fileName$ - right$ (fileName$, 4)

selectObject: "Manipulation " + name$ + "_wav"

tempstr$= "PitchTier " + name$ + "_" + string$(new_Fs)

#writeInfoLine: tempstr$

#plusObject: "PitchTier T1_1000ms__44100"

plusObject: tempstr$

Replace pitch tier

selectObject: "Manipulation " + name$ + "_wav"

Get resynthesis (LPC)

#Scale peak... 0.75

Scale intensity: 65

#To Sound (hum)

#--write to new .wav file-----

#--removes .wav extension-----

samp$= "vowel"

Write to WAV file... 'input_directory$'\'name$'_'samp$'.wav

endfor

#---[EOF]----

# Tables

# Table S1.

Results from the model comparisons regarding Phase, Factor, and Tone.

| Model | Df | AIC | BIC | logLik | Deviance | χ^2^ | Df | Pr(>Chisq) |
| --- | --- | --- | --- | --- | --- | --- | --- | --- |
| Model 1: ~ Phase + (1\|subject) | 4 | 78278 | 78314 | -39135 | 78270 |  |  |  |
| Model 2: ~ Phase + Factor+(1\|subject) | 7 | 78282 | 78345 | -39134 | 78268 | 2.04^a^ | 3 | .565 |
| Model 3: ~ Phase+ Phase:Factor +  (1\| subject) | 13 | 78280 | 78398 | -39127 | 78254 | 15.29^b^ | 9 | .083 |
| Model 4: ~ Phase + Tone +  (1\| subject) | 7 | 76277 | 76340 | -38132 | 76263 | 2006.8^c^ | 3 | <.001 |
| Model 5: ~ Phase * Tone +  (1\| subject) | 13 | 76259 | 76377 | -38117 | 76233 | 29.535^d^ | 6 | <.001 |
| Model 6: ~ Phase * Tone + music + (1\|subject) | 14 | 76244 | 76371 | -38108 | 76216 | 16.993^e^ | 1 | <.001 |
| Model 7: ~ Phase * Tone + music +  (1\| subject) + (1\|item) | 15 | 72293 | 72429 | -36132 | 72263 | 3953.1^f^ | 1 | <.001 |
| Model 8: ~ Phase * Tone + Factor: Tone +music +  (1\| subject) + (1\|item) | 27 | 72284 | 72529 | -36115 | 72230 | 33.04^g^ | 12 | <.001 |
| Model 9: ~ Phase * Tone + Factor: Tone +Phase:Tone:Factor+  music +(1\| subject) + (1\|item) | 51 | 72299 | 72761 | -36099 | 72197 | 33.08^h^ | 24 | .102 |

The Chi square test shows the comparisons of model fit between ^a^the first model and the second model, ^b^the first model and the third model, ^c^the first model and the fourth model, and ^d^the fourth model and the fifth model, ^e^the fifth model and the sixth model, ^f^the sixth model and the seventh model, ^g^the seventh model and the eighth model, and ^h^the eighth model and the ninth model.

# Table S2.

Pairwise comparisons for the effects of Acoustic Factor and Tone.

| Pairwise comparisons | Estimate | Std.Error | *z* | *p* |
| --- | --- | --- | --- | --- |
| **Tone 1** |  |  |  |  |
| Duration-Speaker sex | 0.24 | 0.16 | 1.50 | 0.796 |
| F0 expansion-Speaker sex | -0.15 | 0.16 | -0.91 | 1.000 |
| F0 expansion-Duration | -0.39 | 0.16 | -2.40 | 0.098 |
| Syllable-Speaker sex | 0.13 | 0.16 | 0.82 | 1.000 |
| Syllable-Duration | -0.11 | 0.16 | -0.70 | 1.000 |
| Syllable-F0 expansion | 0.28 | 0.16 | 1.74 | 0.488 |
| **Tone 2** |  |  |  |  |
| Duration-Speaker sex | 0.10 | 0.16 | 0.61 | 1.000 |
| F0 expansion-Speaker sex | -0.09 | 0.16 | -0.58 | 1.000 |
| F0 expansion-Duration | -0.19 | 0.16 | -1.19 | 1.000 |
| Syllable-Speaker sex | -0.02 | 0.16 | -0.10 | 1.000 |
| Syllable-Duration | -0.11 | 0.16 | -0.72 | 1.000 |
| Syllable-F0 expansion | 0.08 | 0.16 | 0.49 | 1.000 |
| **Tone 3** |  |  |  |  |
| Duration-Speaker sex | 0.08 | 0.16 | 0.49 | 1.000 |
| F0 expansion-Speaker sex | -0.15 | 0.16 | -0.91 | 1.000 |
| F0 expansion-Duration | -0.23 | 0.16 | -1.39 | 0.994 |
| Syllable-Speaker sex | -0.05 | 0.16 | -0.31 | 1.000 |
| Syllable-Duration | -0.13 | 0.16 | -0.81 | 1.000 |
| Syllable-F0 expansion | 0.10 | 0.16 | 0.61 | 1.000 |
| **Tone 4** |  |  |  |  |
| Duration-Speaker sex | 0.01 | 0.16 | 0.04 | 1.000 |
| F0 expansion-Speaker sex | -0.10 | 0.16 | -0.64 | 1.000 |
| F0 expansion-Duration | -0.11 | 0.16 | -0.67 | 1.000 |
| Syllable-Speaker sex | -0.15 | 0.16 | -0.95 | 1.000 |
| Syllable-Duration | -0.16 | 0.16 | -0.98 | 1.000 |
| Syllable-F0 expansion | -0.05 | 0.16 | -0.30 | 1.000 |
| **F0 expansion** |  |  |  |  |
| Tone 1-Tone 2 | 0.51 | 0.13 | 3.77 | .001 |
| Tone 1-Tone 3 | 1.15 | 0.14 | 8.47 | <.001 |
| Tone 1-Tone 4 | 0.47 | 0.13 | 3.46 | .003 |
| Tone 2-Tone 3 | 0.64 | 0.13 | 4.74 | <.001 |
| Tone 2-Tone 4 | -0.04 | 0.13 | -0.30 | 1.0000 |
| Tone 3-Tone 4 | -0.68 | 0.13 | -5.03 | <.001 |
| **Duration** |  |  |  |  |
| Tone 1-Tone 2 | 0.71 | 0.14 | 5.22 | <.001 |
| Tone 1-Tone 3 | 1.31 | 0.14 | 9.66 | <.001 |
| Tone 1-Tone 4 | 0.75 | 0.14 | 5.52 | <.001 |
| Tone 2-Tone 3 | 0.60 | 0.13 | 4.49 | <.001 |
| Tone 2-Tone 4 | 0.04 | 0.13 | 0.31 | 1.0000 |
| Tone 3-Tone 4 | -0.56 | 0.13 | -4.17 | <.001 |
| **Speaker sex** |  |  |  |  |
| Tone 1-Tone 2 | 0.56 | 0.13 | 4.18 | <.001 |
| Tone 1-Tone 3 | 1.15 | 0.13 | 8.50 | <.001 |
| Tone 1-Tone 4 | 0.51 | 0.13 | 3.79 | <.001 |
| Tone 2-Tone 3 | 0.58 | 0.13 | 4.36 | <.001 |
| Tone 2-Tone 4 | -0.05 | 0.13 | -0.38 | 1.0000 |
| Tone 3-Tone 4 | -0.64 | 0.13 | -4.72 | <.001 |
| **Syllable** |  |  |  |  |
| Tone 1-Tone 2 | 0.71 | 0.13 | 5.27 | <.001 |
| Tone 1-Tone 3 | 1.33 | 0.13 | 9.84 | <.001 |
| Tone 1-Tone 4 | 0.79 | 0.13 | 5.89 | <.001 |
| Tone 2-Tone 3 | 0.62 | 0.13 | 4.62 | <.001 |
| Tone 2-Tone 4 | 0.08 | 0.13 | 0.63 | 1.0000 |
| Tone 3-Tone 4 | -0.53 | 0.13 | -3.98 | <.001 |

*glmer[correct ~ Phase * Tone +* *Factor: Tone + music +(1| subject) + (1|item), family=”binomial”]. The p values are Bonferroni adjusted.*

# Table S3.

Results from the model comparisons regarding F0 expansion and Tone.

| Model | Df | AIC | BIC | logLik | Deviance | χ^2^ | Df | Pr(>Chisq) |
| --- | --- | --- | --- | --- | --- | --- | --- | --- |
| Model 1: ~ Expansion + music+ (1\|subject) | 5 | 78390 | 78435 | -39190 | 78380 |  |  |  |
| Model 2: ~ Expansion + Tone + music+ (1\|subject) | 8 | 76393 | 76466 | -38189 | 76377 | 2002.5^a^ | 3 | <.001 |
| Model 3: ~ Expansion * Tone + music+ (1\|subject) | 14 | 76291 | 76417 | -38131 | 76263 | 114.68^b^ | 6 | <.001 |

The Chi square test shows the comparisons of model fit between ^a^the first model and the second model, and ^b^the second model and the third model.

# Table S4.

Results from the model comparisons regarding Duration and Tone.

| Model | Df | AIC | BIC | logLik | Deviance | χ^2^ | Df | Pr(>Chisq) |
| --- | --- | --- | --- | --- | --- | --- | --- | --- |
| Model 1: ~ Duration + music+ (1\|subject) | 5 | 77379 | 77424 | -38684 | 77369 |  |  |  |
| Model 2: ~ Duration + Tone + music+ (1\|subject) | 8 | 75348 | 75420 | -37666 | 75332 | 2037.1^a^ | 8 | <.001 |
| Model 3: ~ Duration * Tone + music+ (1\|subject) | 14 | 73146 | 73272 | -36559 | 73118 | 2214.1^b^ | 6 | <.001 |

The Chi square test shows the comparisons of model fit between ^a^the first model and the second model, and ^b^the second model and the third model.

# Table S5.

Results from the model comparisons regarding Speaker sex and Tone.

| Model | Df | AIC | BIC | logLik | Deviance | χ^2^ | Df | Pr(>Chisq) |
| --- | --- | --- | --- | --- | --- | --- | --- | --- |
| Model 1: ~ Speaker sex + music+ (1\|subject) | 4 | 78392 | 78428 | -39192 | 78384 |  |  |  |
| Model 2: ~ Speaker sex + Tone + music+ (1\|subject) | 7 | 76396 | 76459 | -38191 | 76382 | 2002.3^a^ | 3 | <.001 |
| Model 3: ~ Speaker sex * Tone + music+ (1\|subject) | 10 | 76352 | 76442 | -38166 | 76332 | 49.993^b^ | 3 | <.001 |

The Chi square test shows the comparisons of model fit between ^a^the first model and the second model, and ^b^the second model and the third model.

# Table S6.

Results from the model comparisons regarding Syllable and Tone.

| Model | Df | AIC | BIC | logLik | Deviance | χ^2^ | Df | Pr(>Chisq) |
| --- | --- | --- | --- | --- | --- | --- | --- | --- |
| Model 1: ~ Syllable + music+ (1\|subject) | 5 | 78255 | 78301 | -39123 | 78245 |  |  |  |
| Model 2: ~ Syllable + Tone + music+ (1\|subject) | 8 | 76254 | 76327 | -38119 | 76238 | 2007.1^a^ | 3 | <.001 |
| Model 3: ~ Syllable * Tone + music+ (1\|subject) | 14 | 75530 | 75657 | -37751 | 75502 | 735.87^b^ | 6 | <.001 |

The Chi square test shows the comparisons of model fit between ^a^the first model and the second model, and ^b^the second model and the third model.
